# Review on dry reforming of methane, a potentially more environmentally-friendly approach to the increasing natural gas exploitation

**DOI:** 10.3389/fchem.2014.00081

**Published:** 2014-11-11

**Authors:** Jean-Michel Lavoie

**Affiliations:** Département de Génie Chimique et de Génie Biotechnologique, Faculté de Génie, Université de SherbrookeSherbrooke, QC, Canada

**Keywords:** dry reforming, syngas, heterogeneous catalyst, plasma, electricity

## Abstract

With the actual growth of the natural gas industry in the US as well as the potential and availability of this non-renewable carbon source worldwide, reforming of methane gas is getting increasing attention. Methane can be used for the production of heat or electricity, as well, it can be converted to syngas, a building block that could lead to the production of liquid fuels and chemicals, a very promising pathway in light of the increasing price of oil. Amongst the different reforming techniques, dry reforming could represent a very interesting approach both to valorize a cheap source or carbon (CO_2_) as well as to reduce the overall carbon footprint of the increasing worldwide fossil-based methane consumption. In this short review, attention will be given to the thermodynamics of dry reforming followed by an investigation on dry reforming using heterogeneous catalyst by focusing on the most popular elements used in literature for dry reforming. Attention will as well be given to other emerging techniques that may allow countering at one point the high thermodynamic penalties that accompanies conversion of methane using carbon dioxide.

## Introduction

Natural gas is actually one of the cheapest sources of energy available on the planet with an actual market price in the range of 4.4USD/GJ (Jan 2014). A large fraction of the natural gas being used across the globe is actually for the production of heat and electricity. One of the most important consumers in the world is the United States, with an annual consumption of 25,533 billion cubic meters in 2012. The US consumption accounted for 21.27% of the reported world's consumption of 120,017 billion cubic meters that year, rising from the 20.5% of total world consumption the previous year. The natural gas proven reserves were estimated in the US to be 308 trillion cubic feet, approximately 4.5% of the total world reserve of 6846 trillion cubic feet. The countries reported to have the largest confirmed reserved of natural gas are Russia and Iran with 1688 and 1187 trillion cubic meters respectively. While reserves are reported to decrease in Europe, stocks tend to increase year after year in the US, mostly in parallel with shale gas exploitation getting increased attention. Production is as well increasing having been reported to be 20,159 billion cubic feet in 2008 then reaching 24,063 billion cubic meters in 2012, a net increase of about 19%^1^. In close relationship with this tendency toward increasing the world consumption of natural gas, the amount of CO_2_ produced from this fossil fuel is as well increasing year after year. Ranging from 2007 to 2012, the CO_2_ emissions from the consumption and flaring of natural gas went from 6011 to 6754 million metric tons per year worldwide, an increase of about 12% over 6 year, therefore an average of 2% per year increase on CO_2_ emissions related to this fossil fuel. The most significant increases in terms of natural gas consumption and therefore CO_2_ emissions were reported in China were an increase of 86% was observed between 2007 and 2011. Egypt as well showed an increased of CO_2_ production from natural gas in the magnitude of 63% with a major boost in 2008 which is consistent with the period where Egypt started importing oil. Production of CO_2_ related to natural gas increased by 17% in Russia and close to 28% in Mexico. Meanwhile, the amount of carbon dioxide produced from natural gas decreased in Italy and in Germany. In all cases, natural gas is closely related to carbon dioxide, utilization of both carbon-based feedstock is therefore of the essence, one being the most oxidized form of carbon whilst the second present carbon on its most reduced form. In all cases as well, the foreseeable future does not show a tendency toward reducing the consumption of these two compounds. In that sense, chemical engineering should be involved in finding sustainable solutions to ease the impact that both compounds could have long term on the environment. Between 1990 and 2010, a total of 13 thousands wells were drilled in the Barnett fields in Texas (US) thus, once again, supporting the concept of a shale gas revolution in the US (Wang et al., 2014a), a trend that may affect North America entirely in the upcoming years.

Methane can be used directly for the production of heat, electricity or it can be used for the production of syngas via processes as reforming thus generating synthesis gas (syngas). As far as the production of heat or electricity is concerned, if the target is to reduce the carbon footprint generated from the utilization of methane, the target would thus be aimed at the efficiency of the combustion processes. The increasing volume of methane being consumed each year is as well stimulating the research being performed in that field. Najjar showed in a review in 2001 that the engine efficiency was increasingly, reaching theoretical optimum. He showed that the efficiency of engines climbed from 63% of theoretical cycle efficiency in the 1980's to up to 82% of theoretical efficiency near the year 2000. Overall the engines as the ICAD-HAT cycles were reported to reach an overall efficiency reaching 65% (based on % of LHV) (Najjar, 2001).

Production of syngas out of methane can be done using different approaches from partial oxidation reforming to autothermal reforming systems and from steam reforming to dry reforming processes. In all cases the process uses an oxidizing agent that will oxidize methane to carbon monoxide whilst producing hydrogen in a ratio that will vary depending on the type of oxidant used. Autothermal Reforming System (ATR) are reforming systems that aims at the exothermic oxidation of methane with oxygen. However, using pure oxygen comes with a share of technical and economical problems so that the industrial scale systems usually uses a mixture of oxygen, steam and/or CO_2_ with methane. Partial or total oxidation of methane using O_2_ is in all cases an exothermic process, which is a crucial aspect of the ATR systems. Even though the production of syngas is thermodynamically advantaged for a system that would use only oxygen, the economical impact of using only pure oxygen may lead to a combined utilization of CO_2_ and H_2_O in the process. Furthermore, adding CO_2_ or H_2_O to the feed also impact the H_2_/CO ratio in the output gas thus allowing an easier adaptation to many downstream processes including ammonia and methanol synthesis. The reactions mainly involved in the partial to total oxidation of methane using oxygen are depicted below:

(1) $$CH_{4}+2O_{2}=CO_{2}+2H_{2}O\text{ ​​}(\Delta H_{298K}=803,03kJ/mol)$$

(2) $$CH_{4}+O_{2}=CO_{2}+2H_{2}\;\;(\Delta H_{298K}=-322kJ/mol)$$

(3) $$CH_{4}+\frac{1}{2}O_{2}=CO+2H_{2}\text{ ​​}(\Delta H_{298K}=-38kJ/mol)$$

All reactions involving oxygen are exothermic thus thermodynamically favorable starting from the complete oxidation of methane LHV shown in Equation (1), which generates significant amount of heat. When partial oxidation is targeted, the optimal conversion of methane is usually achieved at high temperature. In that sense, and although reactions (2) or (3) may be the targeted one, in most cases it is unavoidable to sacrifice both carbon monoxide and hydrogen by oxidizing them to carbon dioxide and water respectively in order to generate the temperature range required to obtain optimal conversions whilst avoiding the production of soot. Therefore, part of the methane feed is usually invested to generate heat in order to reach operating temperatures above 65°C allowing optimal conversion of methane to syngas and/or hydrogen. A O_2_/CH_4_ molar feed ratio around 0.57, although it would favor Equation (3), would still completely oxidize part of the methane to carbon dioxide via Equation (1 and 2) as well (Lee et al., 2005). The work reported by Bharadwaj and Schmidt, relies on thermodynamic equilibrium calculations, however, usual operating conditions in a ATR is usually around 1000–1200°C in the conversion zone and around 2000°C in the combustion zone (Bharadwaj and Schmidt, 1995). High temperatures are used to avoid the production of secondary products. They are however more energy-demanding and in that sense more of the methane invested in the process is used for heating. Utilization of a catalyst in that case would essentially be to reduce the energy required to reach high conversions of methane.

As an example of research made on ATR systems, recent work made from Wang et al., showed the influence of adding praseodymium to a nickel catalyst supported on alumina generating conversion close to stoechiometric at a temperature of 850°C with an H_2_/CO ratio slightly below 2.5 (original feed composed of 2:1:1 CH_4_:H_2_O:O_2_ at a WHSV of 240,000 mL/g^*^h (Wang et al., 2014b).

Steam reforming (SMR) generally evolves around processes that are, to a certain extent, comparable to ATR in finality although in that case the oxidizing agent is steam. The energetic balance is different from ATR since the production of steam itself requires an energy investment. Furthermore, the thermodynamics related to steam reforming are as well significantly different from an oxygen-based oxidation process as depicted in Equations (4–6) below.

(4) $$CH_{4}+H_{2}O=CO+3H_{2}\;(\Delta H_{298K}=+205kJ/mol)$$

(5) $$CH_{4}+2H_{2}O=CO_{2}+4H_{2}\;\;(\Delta H_{298K}=+163kJ/mol)$$

(6) $$CO+H_{2}O=CO_{2}+H_{2}\;(\Delta H_{298K}=-41kJ/mol)$$

In contrast to oxidizing with oxygen, all reactions involving methane and water are endothermic. An excess of water Equation (5) will lead to a complete oxidation of carbon and the sole production of hydrogen whilst equimolar quantities of both compounds will lead to syngas with a H_2_/CO ratio of 3. Such ratios are rather different from the one obtained when oxygen is used as oxidizing agent (see Equation 1). SMR systems are generally operated at lower temperature with regards to ATR systems but in most of the cases using a nickel catalyst. The steam reforming process are thus divided in 4 steps, the first one being the primary reformer operating at a temperature around 900°C with pressure around 15–30 atm. The latter is then followed by a secondary reformer, also working with a nickel-based catalyst at a average temperature of 1000°C and using a combination of oxygen and steam in the feed. The two following unit operations are generally used to adapt the H_2_/CO ratio using successively a first water-gas shift reaction operated at 400°C under an iron catalyst followed by a second water-gas shift system operated at 200°C under a copper catalyst (Bharadwaj and Schmidt, 1995).

Oxidation of methane for the production of syngas has gone so far from a more oxidizing agent (oxygen) to a lesser one (steam) with pros and cons for each of the approaches. Carbon dioxide can as well be considered as an oxidizing agent via a reaction called dry reforming. This reaction see Equation (7) involves the most oxidized form of carbon (CO_2_) combined with its most reduced form (CH_4_).

(7) $$CH_{4}+CO_{2}=2CO+2H_{2}\;(\Delta H_{298K}=+247kJ/mol)$$

Thermodynamics for the DR reaction are not as favorable as the ATR or even the SMR reactions. However, the proportional consumption of one mole of carbon dioxide per mole of methane invested could reduce the carbon impact, which could overall lead to a “greener” consumption of natural gas (and methane by association). The thermodynamic barrier is however a big challenge and unfavorable to processes that would rely simply on heat to convert the two molecules at high conversion levels. Furthermore, DR also require a pure and steady source of carbon dioxide which may not necessarily be available in all industrial facilities. The use of catalytic systems may lead to a reduction of the energy invested in the process which would downstream allow dry reforming to get closer to an economical process.

In order to assess the potential of an eventual dry reforming technology, a good point of comparison would the SMR reactions. The latter involves processes that are industrially mature, although the reaction is endothermic. Overall the reaction requires +205 kJ/mol over the −803.3 kJ/mol LHV of methane, which is about 25.5% of the energy value of methane. Therefore, a process that would aim in that range of energy consumption for dry reforming would be competitive to the SMR thus scalable to industrial scale. The objective of this review is to investigate on the catalytic systems that were recently reported to lead to high-level conversion both for methane and carbon dioxide. In order to define the limits of the DR, the first section will be dedicated to a study of the thermodynamics of the main and secondary reactions. The second part of this manuscript will be focusing on classical heterogeneous catalyst. Finally attention will be given to other techniques that may diverge from classical system, involving but not limited to plasma or electricity.

## Thermodynamics and equilibrium

The thermodynamics of two important aspects of the dry reforming reaction has to be considered in order to grasp efficiently the possibilities as well as the restrictions of this process. Interactions between carbon dioxide and methane is of course a priority, however, one of the most limiting aspects of the dry reforming reaction is the formation of carbon and the latter should as well be taken into account. The main DR reaction is the direct reaction of CO_2_ and methane as represented by Equation (7). As it was previously indicated, it is the most endothermic reforming reaction that could be considered for reforming methane. Such observation is of course correlated by the fact that carbon dioxide is very stable, completely oxidized and thus getting this molecule to interact, as an oxidant requires significant energy to activate it. Recently, Nikoo and Amin (2011) reviewed the thermodynamics of the different reactions that could be involved in DRM (see Table 1 below).

**Table 1:** **Gas phase reactions in CO_2_ reforming of methane (adapted from Nikoo and Amin, 2011)**.

**Rxn number**	**Reactions**	**ΔH_**298**_ (kj/mol)**
8	*CO*_2_ + *H*_2_ ↔ *CO* + *H*_2_*O*	41
9	2*CH*_4_ + *CO*_2_ ↔ *C*_2_*H*_6_ + *CO* + *H*_2_*O*	106
10	2*CH*_4_ + 2*CO*_2_ ↔ *C*_2_*H*_4_ + 2*CO* + 2*H*_2_*O*	284
11	*C*_2_*H*_6_ ↔ *C*_2_*H*_4_ + *H*_2_	136
12	*CO* + 2*H*_2_ ↔ *CH*_3_*OH*	−90.6
13	*CO*_2_ + 3*H*_2_ ↔ *CH*_3_*OH* + *H*_2_*O*	−49.1
14	2*CH*_3_*OH* ↔ *CH*_3_*OCH*_3_ + *H*_2_*O*	−37
15	*CH*_3_*OCH*_3_ + *CO*_2_ ↔ 3*CO* + 3*H*_2_	258.4
16	*CH*_3_*OCH*_3_ + *H*_2_*O* ↔ 2*CO* + 4*H*_2_	204.8
17	*CH*_3_*OCH*_3_ + 3*H*_2_*O* ↔ 2*CO*_2_ + 6*H*_2_	136
18	*CO*_2_ + 4*H*_2_ ↔ *CH*_4_ + 2*H*_2_*O*	−165
19	*CO* + 3*H*_2_ ↔ *CH*_4_ + *H*_2_*O*	−206.2

All the reactions presented in Table 1 are temperature-dependent and more complex molecules as dimethyl ether should be less favored at high temperature. Therefore, equations (12), (13) and (14) leading to methanol or dimethyl ether, although they are exothermic, would not be favored at temperatures at which dry reforming is usually performed (in the range of 650–1000°C). Therefore, at high temperatures, reaction as reaction 15 and 16 (Table 1) would be more inclined to occur. The same goes for the reactions leading to ethane or ethylene [rxn 8 and 9] despite the fact that alkanes and alkenes have often been reported as side products from dry reforming reactions. At high temperature, reactions that could be observed under dry reforming conditions would most probably be related to reactions with hydrogen as the reverse water-gas shift Equation (8) and methanation reactions Equations (18) and (19).

Nikoo and Amin also provided equilibrium calculations in order to predict the conversion of methane with regards to different CO_2_/CH_4_ ratio at temperatures varying from 300°C up to 1100°C. Their results showed that conversion of methane should be almost complete for oxidizing ratios (CO_2_/CH_4_ of 2 and 3 respectively) starting around 800°C whilst high conversions cannot be obtained below 1000°C when using stoichiometric amounts or below. The comparison between the equilibrium (calculated from Gibbs free energy minimisation) and the actual results was logically dependent on the catalyst that was used, for which the interactions with the gasses were not considered. Despite this fact, the calculation model developed by Nikoo and Amin showed a correlation with a catalyst reported by Khalesi et al. (2008). Finally Nikoo and co-workers studied the effect of pressure at different temperatures for the dry reforming model they developed. According to the latter, the effect of pressure leads to a decrease of the methane and carbon dioxide conversion at all studied temperatures whilst leading to an increase of carbon formation (Nikoo and Amin, 2011). Li et al., also worked on the thermodynamics of dry reforming in comparison with classical steam reforming. Their results showed that conversion of methane should be favored by very high (thus oxidizing) CO_2_/CH_4_ ratios of 5 at low temperatures (750°C). Increasing temperature up to 1000°C should allow maintaining high conversions of CH_4_ for a CO_2_/CH_4_ ratio up to 1 (Li et al., 2008) both observations that were also made by Nikoo and Amin (2011). Wang et al., investigated on the impact of many catalyst and support on the dry reforming reaction especially in the ranges going from 600 to 950°C (Wang et al., 1996). As well, they used equilibrium calculations to investigate on the effect of pressure on conversion as well as on the deposition of carbon on the catalyst.

Since the deposition of coke is related in most studies to the deactivation of the catalyst, it is of outmost importance to identify the reactions that are causing this phenomenon. Table 2 shows the different reactions related to carbon deposition in close relationship with the dry reforming process. The first reaction is known as methane cracking Equation (20), the second is the reverse-Boudouard reaction Equation (21), the last two are the reduction of CO_2_ and the reduction of carbon monoxide, which is both cases produces carbon and water although involving different stoichiometry.

**Table 2:** **Reactions leading to carbon formation linked to CO_2_ reforming of methane (adapted from Nikoo and Amin, 2011)**.

**Rxn number**	**Reactions**	**ΔH_**298**_ (kj/mol)**
20	*CH*_4_ ↔ *C* + 2*H*_2_	74.9
21	2*CO* ↔ *C* + *CO*_2_	−172.4
22	*CO*_2_ + 2*H*_2_ ↔ *C* + 2*H*_2_*O*	−90
23	*H*_2_ + *CO* ↔ *H*_2_*O* + *C*	−131.3

Nikoo and Amin using the same calculation models as previously reported, commented as well on carbon formation. The latter, according to equilibrium calculations, was shown to decrease proportionally with regards to the increasing operation temperature and/or as with the CO_2_/CH_4_ ratio. Calculations using an oxidizing ratio (1 and up) led to a decrease in carbon formation and the latter had a synergetic effect with the increasing temperature. The most oxidizing conditions (as a system where the CO_2_/CH_4_ ratio would be around 3) would lead to no carbon formation above 700°C while for a ratio of 2, the required temperature to avoid carbon formation would be above 750°C. The stoichiometric 1/1 ratio, according to the same model, required temperatures above 1100°C to avoid carbon formation (Nikoo and Amin, 2011). Ginsburg et al., have demonstrated the intimate correlation between the CO_2_/CH_4_ ratio and the formation of carbon on their nickel-based catalyst. The reactant ratio that provided the less carbon accumulation according to their model was the one where the CO_2_/CH_4_ ration was of 2, therefore an oxidizing system where CO_2_ would be in excess (Ginsburg et al., 2005). Li et al., investigated as well on the thermodynamics of the carbon formation during the dry reforming reaction and showed that the latter was very small at high CO_2_ level or at higher temperature. It was shown that the carbon formation obtained from a CO_2_/CH_4_ ratio of 5 at 500°C could be compared to the carbon produced for a stoichiometric CO_2_/CH_4_ ratio at 900°C (Li et al., 2008).

## Heterogeneous catalyst

Dry reforming has been a challenge for chemical engineering for decades. Amongst the publications that were made on the subject, Kokarev et al., were one of the first to report on this subject (Kokarev et al., 1975). From this manuscript published in the 70's, reports on dry reforming increased proportionally yearly reaching 161 in 2013 and increase of 335% in a decade, a tendency that is surely not independent of the increasing interest devoted to natural gas. In terms of catalysts reported to be efficient for this reaction, one of the most commonly reported involves nickel either directly or supported. Table 3 below reviews the operating conditions and the nature of the catalyst used for dry reforming using nickel-based catalysts. Most of these researches shows that utilization of a catalyst can lower the energy demand to activate the reaction with regards to equilibrium calculations. In most of the researches reported as well, conversion levels both for methane and carbon dioxide are generally high. This shows that many of the Ni-based catalysts are efficient to induce DR at lower temperature with regards to what the equilibrium calculations would predict.

**Table 3:** **Dry reforming reactions reported in literature using nickel-based catalyst at different temperatures (T), gaz hourly space velocity (GHSV), with different pressures (P), and reported Time On Stream (TOS)**.

**Catalyst Support**	**Conditions**	**CH_**4**_/CO_**2**_ conversion H_**2**_/CO ratio TOS (h)**	**References**
Ni	*T* = 630°C	76.7%/ND	Ryi et al., 2014
Spinel oxides	GHSV = 23.3 h^−1^	0.80	
	*P* = nd	120 ms	
Ni/Al_2_O_3_	*T* = 550–700°C	78%/90%	Alipour et al., 2014
MgO, CaO, BaO (promoters)	GHSV = 6–18 L/g*h	0.90–0.95	
	*P* = nd	22	
Ni/Al_2_O_3_	*T* = 800°C	90–95%/90–95%	Jiang et al., 2013
Aerogel/xerogel	GHSV = 327 L/g*h	0.95	
	*P* = 1 atm	30	
Ni	*T* = 750°C	95%/95%	Djebarri et al.
Mg, Al, Ce (promoters)	GHSV = 300 L/g*h	1	
	*P* = 1 atm	8	
Ni (plating)	*T* = 700°C	78%/nd	Fukuhara et al., 2013
Ferrite type stainless	GHSV = nd	1.04	
	*P* = 0.8 atm	30	
Ni	*T* = 850°C	80%/80%	Vafaeian et al., 2013
ZSM-5	GHSV = 24 L/g*h	1.00	
	*P* = 0.8 atm	24	
Ni	*T* = 600°C	80%/90%	Arbag et al., 2013
Mesoporous	GHSV = nd	0.78	
Alumina	*P* = nd	40	
Mg,W (promoters)			
Ni	*T* = 700°C	75%/75%	Kathiraser et al., 2013
Al_2_O_3_	GHSV = nd	0.95–0.99	
La (promoter)	*P* = nd	30	
Ni	*T* = 800°C	90%/nd	Chen et al., 2013
CeAlO_3_−Al_2_O_3_	GHSV = 20 L/g*h	nd	
	*P* = 1 atm	20	
Ni	*T* = 700°C	40%/70%	Odedairo et al., 2013
CeO_2_	GHSV = 38.4 L/g*h	0.55	
	*P* = 1 atm	4.2	

However, despite high conversion rates observed for many nickel catalyst, the major problem is carbon deposition. Nickel (as an unsupported metal) also acts as catalyst, especially when the catalyst is promoted by the insertion of different rare earths. In the latter case, the longevity of the catalyst becomes a crucial parameter and one of the most important points of comparison. DR reactions are, if efficiently completed, generating equimolar ratio of carbon monoxide and hydrogen (which can be directly extrapolated to the volumes produced). Thus a sign that the reaction is efficient should combine high conversion of methane as well as a H_2_/CO ratio of 1. If the ratio is somehow higher than one, it means that there is proportionally more hydrogen produced with regards to carbon monoxide. Since pure DR does not involve water, the water-gas shift reaction does not occur significantly and the loss of CO is essentially related to the formation of soot. The latter can occur via two different reactions; methane cracking (Equation 20) and the Boudouard reaction (Equation 21). According to Nikoo and Amin, from 650°C up to 1000°C, the methane cracking reaction is favored over the Boudouard reaction (Nikoo and Amin, 2011). H_2_/CO ratio lower than one would show that there is more carbon monoxide produce with regards to the hydrogen. Such observation could as well be linked to carbon formation via CO_2_ direct reduction with hydrogen (Equation 22). Occurrence of this reaction could be confirmed by the formation of water at the output of the catalytic system. The last reaction that could lead to production of carbon is the direct reduction of carbon monoxide (Equation 23). This reaction is less favored with regards to temperature and is favorable in temperature ranges comparable to the Boudouard reaction. A good indication of the occurrence of this reaction would be a H_2_/CO ratio maintaining around 1 combined with the production of water.

Keeping in mind the fact that the DR reaction is more endothermic than the SMR or the ATR, the required aspect of an optimal catalyst would be to induce high conversions of CH_4_ without however leading to deactivation. Carbon formation can as well be a problem if it does not deactivate the catalyst since it represents a net loss of carbon that has to be taken account of when calculating mass balances. As mentioned earlier, nickel is by far one of the most common metal used for dry reforming and Table 3 summarize different researches that were performed using this metal. Work by de Sousa et al. (Ryi et al., 2014) showed that the nature of the support had a direct impact on the dry reforming reaction. However, the best results were obtained for the catalytic systems that contained Ni^0^ nanoparticles. They also confirmed that both nickel and cobalt led to coke deposition, which in turn led to deactivation of the catalyst. The low H_2_/CO reported could be a sign that soot was formed by reduction of CO_2_ (Equation 22), which is favored by low temperature keeping in mind that the ratio of reactant entering the reactor was equivalent during these experiments. Alipour et al. (2014) tested the effect of different alkaline-earth metal oxides on a alumina-supported nickel catalyst. The latter have investigated the effect of the catalyst over different parameters including temperature, time on stream and CH_4_/CO_2_ feed ratio. Results shows that conversion of methane increased with temperature for all catalyst reaching around 80% both for the nickel catalyst alone or the latter promoted by magnesium. CO_2_ however shows better conversion reaching 90% for the magnesium oxide promoted system, close to 10% more than the catalyst without promoter. Combined to this observation, the H_2_/CO ratio increased with regards to temperature starting a 0.70 à 500°C and reaching close to 0.93 at higher temperatures. Such an observation could be a sign that either CO_2_ react with the catalyst or the promoter in some case or that there might be formation of soot from hydrogenation of CO_2_.

The reaction could as well be a partial reduction of CO_2_ promoted by the MgO. Overall, all systems were shown to be maintaining during the total operating time of 300 min whilst the catalyst that had the smallest crystallites were shown to be the most efficient for the reaction.

Conversion decreased significantly with regards to the GHSV with the best results were observed below 6 L/g^*^h. Finally the best reported CH_4_ conversion were observed for a 4/1 ratio in favor of CO_2_ whilst a 1/1 ratio led to an even conversion of 60% for both reactants. The size of the particle is as well a parameter that was investigated by Jiang et al., who studied the effect of having a alumina supported nickel catalyst on aerogel or on xerogel form (Jiang et al., 2013). In this work, as for Alipour et al. (2014), alumina was used as support although in that case the total nickel loading was of 20%, twice what was used by Alipour et al.,. Their results shows that the aerogel Ni on alumina catalyst was maintaining activity after 30 h (although decreasing slightly) while the xerogel was starting to deactivate right after the first hour of operation. One of the reasons that could explain such behavior is related to the physicochemical properties of both catalyst and in that sense, the aerogel showed higher specific surface, a lower bulk density, a lower crystallite size and a higher Ni dispersion as compare to the xerogel. Djebarri et al., worked with nickel combined either with cerium, magnesium or aluminum (Djebarri et al.). Best conversions for methane were achieved with the NiMgAl and the NiCe catalyst which both achieved a steady conversion of methane of 95 and 85% respectively over 8 h of operation. As well, both catalysts were generating a H_2_/CO ratio between 0.95 and 1.0, which is a good sign that secondary reaction did not occur. If correlated with the crystallite size, both catalysts that showed the optimal effect had the smallest crystallite size after reduction. The NiMgAl crystallites were evaluated to be of 17 nm as for the NiCe catalyst, crystallite size were of 26 nm. Such tendency tend to support that methane has to interact with nickel in order to react although the amount of available sites should be limited to avoid interaction between the adsorbed methane molecules. Fukuhara et al., worked as well with a nickel based catalyst that they impregnated on a honeycomb-like structure (Fukuhara et al., 2013). They tried impregnated nickel on the surface of the honeycomb structure using an electroless technique. Results shows that the catalyst produced by the electroless technique was more efficient at high temperature as well as more durable (less deactivation) than the commercial catalyst. Best conversions were obtained at 700°C generating a H_2_/CO ratio close to the unity leading to the assumption that secondary reactions are limited. The commercial catalyst, used under the same condition has generated H_2_/Co ratio above 1 (1.42) showing a significant carbon deposition in the process.

The work reported by Vafaeian et al. (2013) showed the impact of using ultrasounds during the impregnation of a zeolite support. The authors used different concentrations of nickel ranging up to 20% mass of the catalyst. Overall, the activity of all catalyst grew in regards to temperature increments. However, they showed as well that the larger concentrations of Nickel were not beneficial for the dry reforming reactions. The results peaked around 8% content of nickel in the zeolite, reaching a conversion for both methane and CO_2_ in the magnitude of 80%. Under strict dry reforming reactions, the H_2_/CO ratios closest to stoichiometry were obtained for a catalyst that used a 3% nickel content. A 8% nickel content has generated a H_2_/CO ratio below the unity (showing a possible side-reaction of hydrogen). Arbag et al., also investigated on different methods to prepare their catalyst, in order to optimize the interactions between the reactant and the metal sites without however inducing carbon formation (Arbag et al., 2013). They tried impregnation on mesoporous alumina that was produced either via a hydrothermal process or with sol-gel. Nickel salts were then impregnated on the material using ethanol as solvent. The following dry reforming reactions were performed at 600°C and the catalyst that showed the best effect on methane conversion was the nickel supported on the mesoporous alumina produced using the sol-gel approach. Conversion for methane was around 80% whilst CO_2_ was around 90%. Addition of other metals (tungsten and magnesium) did not have a positive impact on the reaction (conversion rates declined) whilst there was no visible impact on the deactivation of the catalyst. Kathiraser et al., used a classical wet impregnation technique to produce three nickel-based catalyst (Kathiraser et al., 2013). Whilst the first was classical (nickel on γ-alumina) the other two involved lanthanum either as metal (NLA) or as oxide (NL). The first test on stream that the authors did was at 700°C and results showed that although most of the catalyst allowed conversion of 65% methane, the nickel/lanthanum catalyst was the one the was deactivating the slowest. The nickel on lanthanum oxide (Ni/La_2_O_3_) was the least effective catalyst both for CO_2_ and for CH_4_ conversion leading to the conclusion that the alumina does play a role in the reaction. The combined nickel/lanthanum catalyst led to a produced H_2_/CO ratio close to the unity whilst the other two catalysts were closer to 0.9. One of the observations that could explain such phenomenon, for the nickel on gamma alumina catalyst the conversion ratio for CO_2_ is higher than methane, which can be a sign that hydrogen produced from the reaction is reducing CO_2_ to CO thus reducing de H_2_/CO ratio significantly below unity. Increasing the temperature to 800°C as well led to an increase in conversion both for the nickel-alumina and the nickel/lanthane on aluminum oxide catalyst. Results shows in both case an increase in methane conversion going up to 85%. CO_2_ however is above 90%, which should lead to H_2_/CO ratio again significantly below unity (not mentioned in the manuscript). The nickel/lanthanum catalyst although it showed comparable efficiency as the NA catalyst, maintained steady conversion for 35 h leading to the assumption that La may have increased the durability of the catalyst. Finally, Kathiraser et al., showed that the particle size for the most durable catalyst were significantly smaller than the particles for the classical nickel on gamma-alumina. Chen et al., worked as well with a nickel on alumina catalyst (Chen et al., 2013), however, they added cerium in the basic alumina structure at concentrations varying between 0 to 15%. Long-term tests, performed at 800°C showed that the catalyst without cerium was slightly more efficient at conversion of both methane and CO_2_. The different catalysts produced from varying concentrations of cerium were in most case very comparable as for conversion of the reactants. However, the authors also quantified the carbon produced during the long-term process and they evaluated as well the carbon deposition of the different catalyst. They were able to demonstrate that the cerium concentration had an impact inversely proportional to the amount of cerium in the catalyst. Therefore, the catalyst that had 15% cerium had a carbon content of 0.29 g/g post-reaction whilst the catalyst without any cerium had 0.92 g/g of carbon deposition. Thus, despite the fact that most of the catalyst allowed a conversion of methane up to 80% (at 800°C) the utilization of cerium may have an impact on the catalyst longevity, which could be an asset under industrial conditions. Odedairo et al., also used cerium in their catalyst more specifically cerium oxide that was used as support on which nickel was impregnated via classical wet impregnation (Odedairo et al., 2013). They prepared the cerium oxide particles via two pathways, one thermal and the other using plasma. The test were performed for more than 10 h (700 min) at 700°C and results showed that the catalyst for which the support was produced by plasma deactivate significantly more slowly as compared to the one produced thermally. The H_2_/CO ratio for both catalytic system were significantly below unity (0.58 and 0.48 for the plasma and the thermally produced catalyst respectively). This tendency can be related to the fact that overall more carbon dioxide was converted than methane, which can only be a sign of CO_2_ reduction with the hydrogen produced from methane cracking (see Equations 20 and 8).

From Table 3, it can be observed that the highest conversions were related to the size of the nickel particles on the catalyst. Sousa et al. (Ryi et al., 2014) used nickel nanoparticles for their catalyst whilst Jiang et al., used aerogel and xerogel for the production of their catalyst (Alipour et al., 2014). In both cases the size of the crystallite was shown to have a significant importance on the conversion as well as the durability of the catalyst in time. The size of the active crystallite was as well an important factor for Djebarri et al., where the most efficient catalyst was the one that had the smallest crystallite size. Fukuhara et al., were able to deposit nickel particle on an honeycomb-type structure (Fukuhara et al., 2013) and for them as well, the catalyst production system led to small particles that were very efficient as well as durable for the DRM. The utilization of ultrasound permitted as well a good separation of the nickel particles on the catalyst surface for Vafaeian et al. (2013) which overall provided good conversion results. Numerous references published in Table 3 mentioned that the concentration of nickel in the catalyst was a fairly important factor. The optimal concentration seemed to vary between 7 and 10% wt. of the catalyst. Utilization of a promoter or a co-catalyst did not provide higher conversion results although in some cases it allowed a reduction of carbon formation, which is a very important asset for the DRM catalyst. Utilization of cerium or lanthanum was reported as efficient to this purpose whilst metals as magnesium or tungsten were not. The conclusion that could be drawn from an overview of these results is that the nickel particle has to be relatively abundant although they have to be as small and as spread as possible on the catalyst. Utilization of a promoter or a co-catalyst was found efficient in such case where the catalyst allowed further spreading of the nickel particle or when it promoted activation of the carbon monoxide. As well, the co-catalyst may have been found efficient in such case where it promoted inhibition of carbon formation. The effect of the co-catalyst is more detailed in Table 4 where the impact of alkali and alkaline earth was investigated with regards to the DRM reaction. Although many metals were investigated in common with nickel for the DRM reaction, the alkali and alkali metals are usually considered as Lewis bases. The availability of the electrons may provoke interactions with the carbon dioxide leading to a semi-carbonate form. The latter, in conjunction with methane cracking that can occur on nickel, would possibly be a good opportunity for the DRM reaction since it would involve activation of both reactants.

**Table 4:** **Dry reforming reactions reported in literature using nickel and a basic element as main catalyst at different temperatures (T), gaz hourly space velocity (GHSV), with different pressures (P), and reported Time On Stream (TOS)**.

**Catalyst**	**Conditions**	**CH_**4**_/CO_**2**_ conversion H_**2**_/CO ratio TOS**	**References**
Ca/Ni/K (2:1:0.1)	*T* = 860°C	95%/95%	Shamsi, 2006
Ca/Ni/Na (2:1:0.1)	GHSV = 5.04	0.9	
	[O] = CO_2_	350 h	
	*P* = 1 atm		
Ni/K-MgO-ZrO_2_	*T* = 750°C	85%/85%	Nagaraja et al., 2011
	GHSV = nd	nd	
	[O] = CO_2_	15 h	
	*P* = 1 atm		
Ni/Al_2_O_3_	T = 750°C	80%/85%	Castro Luna and Iriarte, 2008
K, Sn, Mn, Ca	GHSV = nd	nd	
	[O] = CO_2_	30 h	
	*P* = 0.1 MPa		
Ni/ZrO_2_	*T* = 500–700°C	70%/70%	Rezaei et al., 2008
CeO_2_, La_2_O_3_, K_2_O	GHSV = 15	0.95	
	[O] = CO_2_	50 h	
	*P* = 1 atm		
Ni-(Co,Ca,K,Ba,La,Ce)	*T* = 750°C	53%/60%	Fan et al., 2011
MgO-ZrO_2_	GHSV = 144	nd	
	[O] = CO_2_	40 h	
	*P* = 1 atm		
Ni	*T* = 700°C	57%/67%	Juan-Juan et al., 2006
Al_2_O_3_	GHSV = 22.5	nd	
K (promoter)	[O] = CO_2_	24 h	
	*P* = 1 atm		
Ni	*T* = 650°C	32%/42%	Frusteri et al., 2001
MgO	GHSV = 5.5	nd	
K (promoter)	[O] = CO_2_	12 h	
	*P* = 1 atm		
Ni	*T* = 800°C	85%	Nandini et al., 2005
Al_2_O_3_	GHSV = 2.85	1.07	
K, CeO_2_, Mn (promoters)	[O] = CO_2_	6 h	
	*P* = 1 atm		

Shamsi reported in 2006 on a Ca/Ni/K catalytic system that was shown to be very efficient for the dry reforming reaction whilst as well showed no deactivation after nearly 360 h (Shamsi, 2006). The concentrations of metals in the catalyst were of 2:1:0.1 (Ca/Ni/K) and the system was tested at different temperatures and pressures values. Below 750°C, the conversion of methane reached a maximum of 46% whilst an optimal conversion of 99.5% was reached at 850°C and 1 atm. Increasing the pressure in the reactor led to a drop both in methane and CO_2_ conversion at 800°C. Conversion of methane reached 98.7% at 1 atm whilst decreased to 0.80 at 9 atm and 70 at 14 atmospheres. At 860°C the conversion of both methane and carbon dioxide were 99.5 and 96.3. At higher pressure (14 atm) the conversion increased proportionally to temperature and conversion went from 35% (for methane) at 800°C to 75% at 900°C. In both cases, conversion of carbon dioxide was more important than methane at higher temperature, ranging from 70% at 800°C to 85% at 900°C. Nagaraja et al., studied as well the effect of potassium as a doping agent for their nickel catalyst (Nagaraja et al., 2011). In their case, the support was custom-made out of magnesium and zirconium oxides (MgO-ZrO_2_) to which about 10% wt. of nickel was added. To this mixture, they finally added potassium at mass ratio varying from 0 to 1.9%. The results from the DRM reaction performed from 550 to 750°C showed that the original catalyst without the addition of potassium was one of the most efficient one for the conversion of methane reaching about 90% conversion at high temperature. The original catalyst was bested only by the one doped with a maximum of 0.5% of potassium which was closely following values obtained from equilibrium calculations. Higher concentrations of potassium were shown to inhibit the conversion of methane reaching as low as 65% for the highest temperature investigated in this work. Nagaraja et al., also showed the effect of the different concentration of potassium on longer period tests (ranging up to 16 h). Amongst all the catalysts that were studied, only the one that did not have potassium showed deactivation as soon as the reaction started. All the other catalyst were maintaining their activity whilst the one with a 0.5% concentration of potassium remain the optimal in terms of conversion maintaining at a 90% conversion of methane during the whole period. Luna and Iriarte worked on a classical nickel on alumina catalyst that they prepared using a sol-gel method (Castro Luna and Iriarte, 2008). With this original catalytic structure, the authors tested the effect of adding separately 4 elements (K, Ca, Mn, and Sn) at a 0.5% wt. charge. The original catalyst (nickel on alumina) generated the best conversion results (84.7% for methane) that were maintained for a total of 30 h at 750°C. Their catalyst led to a H_2_/CO ratio of 0.97 although carbon formation was observed (60 mg/g of catalyst). The same catalyst doped with potassium provided lower conversion results (81.3%) and a very low H_2_/CO ratio (0.4). The ratio cannot be correlated to the amount of CO_2_ consumed, which was slightly higher than CH_4_ (86%). However, carbon formation for K-doped catalyst was below 10 mg/g showing. As well for this case as for the previous one, the system was maintained at 750°C for 30 h and for both cases no deactivation was observed for the whole duration of the DRM reaction. Tests on calcium, tin and manganese have generated lower conversion in all cases (except for calcium that showed an increase in conversion after 15 h). All of the catalyst showed deactivation after 30 h. Calcium-doped catalyst provided the closest H_2_/CO ratio to the original nickel on alumina catalyst (0.88) whilst all the other had ratios below 0.45. Amongst all the catalyst tested, the one involving potassium led to the lowest carbon formation (<10 mg/g) whilst the manganese-doped catalyst has generated 250 mg/g of carbon. In all cases, the formation of carbon can be directly correlated to the deactivation pattern observed for the catalyst. Rezaei et al., reported on the utilization of different promoters (CeO_2_, La_2_O_3_, K_2_O) on a nickel catalyst supported on zirconium oxide (Rezaei et al., 2008). The zirconium oxide support was prepared using a surfactant and calcinated at different temperatures, where treatment at 700°C produced the smallest pores inside of the structure. Of all the catalytic mixtures that were tested in this work, 5% Ni on the ZrO_2_ matrix provided the lowest conversions. The catalytic mixture that produced the highest conversions were as well as the 5% Ni involving 3% K_2_O or 3% La_2_O_3_ for which methane was converted in both cases up to 75% at 700°C. The H_2_/CO oscillated around 0.95 in all cases and remained stable for 50 h. Long term tests showed as well a slow but steady deactivation of the catalyst for all system that were tested although the tendency was more pronounced for the nickel on zirconia without any promoter. The catalyst that remained the most stable with regards to time was the one involving 3% La_2_O_3_.

Fan et al., studied the effect of nickel-based bimetallic catalyst one the DRM of methane (Fan et al., 2011). The metals that were investigated are cobalt, lanthanum, cerium, barium, manganese, potassium and calcium all of which were combined with nickel on a MgO-ZrO_2_ support. Experiments were performed at 750°C and 1 atm using a GHSV of 144 L/g^*^h with a feed ratio (CH_4_/CO_2_/N_2_) of 1/1/1. As far as conversion of methane is concerned, highest conversion were obtained with the Ni-Co catalyst (about 82%) whilst the lower conversions were observed for the Ni-Ca catalyst. Most of the catalyst tested showed a slight deactivation after 40 h except for the Ni-K, Ni-Ca, and Ni-Co catalyst, which not only decreased but showed as well a slight increase during the tests (from 75 to 80%). Conversion of CO_2_ was overall more stable than for methane, and as for CH_4_, the best conversions were obtained for the Ni-Co catalyst whilst the lowest were obtained for the Ni-Ca catalyst. Conversion of CO_2_, as for conversion of CH_4_, increased from 80 to 85% during the 40 h testing period using the Ni-Co catalyst. Carbon formation was shown to be higher for the Ni-Mn catalyst followed by the Ni-Ca and the Ni-K catalyst. The authors did correlate this tendency to the change in conversion and showed that the formation of carbon does not necessarily have a bad effect on conversion. The Ni-Mn catalyst showed a strong carbon formation (more than 2 mg/g^*^h) combined with a drop of conversion in the magnitude of 7%. As opposed to this tendency, the Ni-Co catalyst showed a carbon formation a little below 1 mg/g^*^h which was correlated with a 8% methane conversion increment. The occurrence of char particles on the surface of the catalyst can thus be beneficial for the DRM reaction. Juan-Juan et al., tested the effect of a potassium-doped nickel on alumina catalyst that was produced by a simple wet impregnation of gamma-alumina (Juan-Juan et al., 2006). The nickel loading in the catalyst was typically of 7.5% wt. for the Ni/Al_2_O_3_ catalyst and 9% wt. for the NiK/Al_2_O_3_. The results shows, when the system is operated at 700°C, that the conversion of methane was similar for both catalyst. The tests were performed for a maximum duration of 6.7 h and there was at this point no evidence of deactivation in both cases. The NiK catalyst led to a lower conversion of carbon dioxide as compared to the classical nickel on alumina catalyst however the conversion of both gasses was very comparable which may lead to a better H_2_/CO ratio. Furthermore, higher conversion of CO_2_ could as well lead to oxidation of the catalyst or part of the catalyst and maintaining the conversion version similar for CO_2_ and CH_4_ may provide a better durability of the catalyst. The authors as well verified the impact of temperature on the conversion of methane and carbon dioxide for both catalysts. The classical nickel on alumina catalyst showed a tendency that could be quite comparable to equilibrium calculations. The work reported as well on temperature programmed reactions (TPR) where the two catalysts were tested at different temperatures and compared to equilibrium calculations. The NiK/Al_2_O_3_ catalyst showed conversions that were below the Ni/Al_2_O_3_ for most of the temperatures investigated (127–827°C) although both catalysts met the equilibrium calculations (for methane conversion) above 627°C. Frusteri et al., investigated the effect of potassium on the stability of a nickel catalyst supported on magnesium oxide (MgO) (Frusteri et al., 2001). The authors tested 3 catalysts, one where nickel was impregnated on the magnesium oxide support solely and the two other where potassium was added with loadings of 1.5 and 2.5% respectively. For all catalyst the nickel loading was close to 20% wt. varying from 17.3 to 19.0% of the catalyst weight. Operations with the catalyst at 650°C showed a better conversion for the catalyst without any potassium. However, conversion declined fast going to 50–25% in 12 h. CO_2_ conversion followed the same tendency although the drop was less important going from 55 to 40% in 12 h. Both catalysts that had potassium content showed a lower conversion, which was inversely proportional to the amount of K added to the catalyst. The catalyst with the least potassium content reached a methane conversion in the magnitude of 30% whilst the other got to 25%. However, in both cases the catalyst remained stable although slowly deactivating for the 12 h duration of the test. The authors as well investigated the impact of the operation on the size of the particles and the catalyst without any potassium showed an increase in particle size with an average of 5.0 nm prior to reaction going up to 10.0 nm in average post-reaction. The catalyst that had the lowest amount of potassium showed an average particle size of 10 nm prior and after the reaction. As well, the rate of carbon deposition was higher for the catalyst without any potassium (100 mg/g^*^h) a value significantly higher than what was observer for the catalyst with 1.5 and 2.5% potassium loading that lead to a carbon deposition of about 5 and 10 mg/g^*^h respectively. This shows that potassium has indeed an impact on durability of the catalyst although it should not be too concentrated in order to avoid carbon deposition and gain higher conversions. Nandini et al., as well worked on a Ni/Al_2_O_3_ catalyst, although in their case they tested the effect of potassium, cerium oxide as well as magnesium oxide (Nandini et al., 2005). The authors have tested different nickel content, ranging from 0 to 18% wt. whilst potassium was investigated at concentrations from 0.5 to 3% wt., CeO_2_ from 0 to 15% and MnO from 0 to 15%. At 800°C, results showed that conversion was increasing proportionally to the nickel content on the alumina support. Conversion of methane reached 90% at a 13.5% loading of nickel and behavior of the catalyst with 18% Ni showed the same tendency under similar experimental conditions. When using a nickel loading of 8.8% wt. and concentrations of potassium varying from 0 to 3%, the authors observed that increasing the potassium content was beneficial for the conversion of methane. The latter increased from 0.5% to an optimum at 2.2% wt. although in the latter case the conversion was quite comparable to the nickel catalyst without any potassium. Although the experiments in this work have been performed for about 6 h, quantifying carbon formation on the catalyst assessed a potential deactivation of the catalyst. Amongst the different catalysts that were tested, the nickel on alumina without any promoter produced the largest quantity of char that occurred proportionally with the Ni loading at 800°C. Adding potassium to the catalyst led to a decrease of the carbon produced proportionally to the amount of potassium. Therefore, the catalyst that had 2.9% of K showed the least amount of carbon produced. At lower temperature (750°C), cerium and magnesium oxide showed to have a very positive effect on coke reduction on the catalyst. The loading of coke was of 3.1% wt. post-reaction for a nickel content of 13.5%, the latter decreased to less the 1% when adding either 10% wt. of cerium oxide or 5% wt. MgO.

Retrospectively, Table 4 showed the effect of adding alkaline or alkaline earth to the classical nickel catalyst. Overall, the results showed that adding Lewis bases to the catalytic mixture did not, in most of the cases, contribute to increase conversion of methane. In most of the cases however, utilization of elements as potassium has allowed to reduce carbon formation, which is important aspect ensuring catalyst durability avoiding deactivation. Results show as well that adding different elements to the catalytic mixture allowed avoiding carbon formation even at low temperature thus inhibiting reactions as methane cracking. Both from Tables 3, 4, results show that nickel is a very efficient element for the dry reforming reaction. However, the latter tends to produce carbon under operating conditions, which leads to catalyst deactivation. Utilization of other elements or oxides can reduce the amount of carbon formation although it often tends to generate lower conversions. The required effect of the catalyst has thus to provoke methane cracking (Equation 20) whilst promoting the removal of coke, using reactions as the reverse Boudouard Equation (21). In Table 5 below are reported the impact of other elements or oxides for the DRM reaction. Laosiripojana and Assabumrungrat reported on the utilization of high and low-surface ceria (CeO_2_) for the DRM reaction at different CH_4_/CO_2_ ratios and compared the performance on Ni/Al_2_O_3_ catalyst (Laosiripojana and Assabumrungrat, 2005). The high-surface area catalyst was shown to allow conversion of methane higher than 32% (900°C). The low-surface ceria that was tested as well under the same conditions led to conversion of methane of about 15%. A CH_4_/CO_2_ ratio of 3 showed the best results for the conversion of methane although not drastically above the 1/1 ratio. When the CH_4_/CO_2_ ratio was below 1, both the high surface (HAS) and the low surface area (LSA) catalyst showed a slightly lower conversion of methane over the 10 h duration of the test. Although the conversions are significantly lower as compared to the classical Ni/Al_2_O_3_ systems, the authors showed that the catalyst remained stabled and did not deactivate as drastically as the Ni/Al_2_O_3_ catalyst. Deactivation was quantified as 6.9% for the high surface area ceria and 30% under dry reforming conditions. Under the same conditions, the low surface area catalyst showed 30% deactivation whilst the classical nickel on alumina catalyst reached a 96% deactivation after 10 h. Carbon formation for the HSA was close to none whilst it was below 0.2% for the LSA as compared to 5.2% for the Ni/Al_2_O_3_ catalyst. However, both ceria catalysts showed surface reduction during operation in dry reforming whilst the Ni/Al_2_O_3_ did not show any. This surface area correlates with the deactivation of the CeO_2_ catalyst in both cases. Brungs et al., studied molybdenum carbide on different supports (Al_2_O_3_, ZrO_2_, SiO_2_, and TiO_2_) for the DRM reaction (Brungs et al., 2000). The best catalyst, evaluated at 946°C and 8 bars with a ratio CH_4_/CO_2_ of 1 was for the Mo_2_C catalyst supported on SiO_2_ where conversion of methane reached 91%. The less efficient combination was shown to be Mo_2_C/TiO_2_ for which methane conversion was reported at 31%. Although both Mo_2_C/SiO_2_ and Mo_2_C/ZrO_2_ lead to higher conversion of methane, the Mo_2_C/γ-Al_2_O_3_ remained the most stable during 40 h of operation with a conversion of methane close to 90%. The support is therefore related to the conversion of methane but the catalyst as well since reduction of the Mo_2_C content led to a decrease of methane conversion for the Mo_2_C/γ-Al_2_O_3_ catalyst whilst as well showing a faster deactivation. Brungs et al., also investigated the effect of the calcination period on the activity and deactivation of the catalyst. The two systems that were investigated for this part of the article were the Mo_2_C/SiO_2_ and Mo_2_C/γ-Al_2_O_3_ catalyst. In both cases, calcination of the catalyst for 24 h lead to conversions as good as for the one calcined for 4 h. However, results show that deactivation was significantly higher for the catalyst that was calcined for 24 h, going from 90 to 10% in 20 h. However, the catalyst that was pretreated only for 4 h remained stable (Mo_2_C/γ-Al_2_O_3_) or only declined slightly (Mo_2_C/SiO_2_).

**Table 5:** **Dry reforming reactions reported in literature using metals (other than nickel) at different temperatures (T), gaz hourly space velocity (GHSV), with different pressures (P), and reported Time On Stream (TOS)**.

**Catalyst**	**Conditions**	**CH_**4**_/CO_**2**_ conversion H_**2**_/CO ratio TOS**	**References**
CeO_2_	*T* = 900°C	32%/nd	Laosiripojana and Assabumrungrat, 2005
	GHSV = 4	0.93	
	[O] = CO_2_	10 h	
	*P* = 1 atm		
Mo_2_C	*T* = 947°C	90%/93%	Brungs et al., 2000
SiO_2_, Al_2_O_3_, TiO_2_, ZrO_2_	GHSV = 2.6	0.95	
	[O] = CO_2_	40 h	
	*P* = 7.9 atm		
Ru	*T* = 750°C	57%/nd	Ferreira-Aparicio et al., 2000
SiO_2_, Al_2_O_3_	GHSV = 120	nd	
	[O] = CO_2_	10 min	
	*P* = nd		
VC, NbC, TaC	*T* = 1050°C	90%/90%	Brungs et al., 1999
	GHSV = 1.2	0.96	
	[O] = CO_2_	25 h	
	*P* = 7.9 atm		
Ru, Rh, Pt	*T* = 850°C	95%/nd	Tsyganok et al., 2004
Mg(Al)O_X_	GHSV = 227	1.8	
	[O] = CO_2_ and O_2_	5 h	
	*P* = 1 atm		
Co	*T* = 800°C	95% + /95% +	Bouarab et al., 2004
MgO-SiO_2_	GHSV = 13	1.12	
	[O] = CO_2_	13 h	
	*P* = 1 atm		
CoNdO_x_	*T* = 850°C	92%/92%	Choudhary et al., 2005
	GHSV = 20	0.91	
	[O] = CO_2_	60 h	
	*P* = 1 atm		
Ir/Ce_0.9_Gd_0.1_O_2−x_	*T* = 800°C	65%/85%	Wisniewski et al., 2005
	GHSV = 30	0.9	
	[O] = CO_2_	20	
	*P* = 1 atm		
Pt	*T* = 700°C	80%/80%	Özkara-Aydınoğlu et al., 2009
ZrO_2_	GHSV = 15.6	1	
Ce (promoter)	[O] = CO_2_	4	
	*P* = 1 atm		

^*^GHSV, Gas hourly space velocity expressed in l/g_cat_^*^h.

Ferreira-Aparicio et al., investigated on the effect of ruthenium catalyst (about 1% wt. content) using either SiO_2_ or γ-Al_2_O_3_ as support (Ferreira-Aparicio et al., 2000). The tests were performed at 550°C and 750°C with an entering feed composed of CH_4_/CO_2_/He (10/10/80) at a flow rate of 100 cm^3^/min. Results performed at low temperatures (550°C) showed nevertheless a conversion of methane in the magnitude of 12 and 14 % after 10 min of operation for the ruthenium on silica and alumina respectively. Increasing the temperature to 750°C led to an increased conversion of methane reaching 57 and 52% for the Ru/SiO_2_ and Ru/Al_2_O_3_ catalyst respectively. As for CO_2_, its conversion was reported to be higher at 550°C with a X_CO2_/X_CH4_ ratio of 1.4 and 1.2 respectively for the silica and alumina support. This shows that under these conditions, CO_2_ has a better interaction with the catalyst as compared to methane. However, the results obtained at 750°C showed that increasing temperature have decreased the gap between the methane and CO_2_ conversion closer to one, more specifically 1.2 when using silica as support and 1.0 when using alumina. The conversion rates also increased with regards to temperature, rising by a 2 factor from 550 to 750°C. Lower temperature led to significant deactivation of the catalyst, 50% after 3 h and 83% after 24 h for the Ru/SiO_2_ catalyst. As for the Ru/Al_2_O_3_, deactivation was less pronounced, loosing about 9% in the first 3 h followed by 40% in 24 h. At higher temperature, the deactivation was shown to be different for the two catalysts. Whilst it was less pronounced after 3 h for the silica support (44%), it was more important for the alumina support (17%). Important aspects of this article show once again that the dry reforming reaction tends to be an equilibrium between methane cracking and CO_2_ reduction. The lower temperature experiments showed a higher conversion of CO_2_ whilst the highest showed equilibrium between CO_2_ and methane consumption. In both cases, the Ru/Al_2_O_3_, which had the best X_CO2_/X_CH4_ ratio was as well the catalyst that deactivated the slowest. Brungs et al., investigated on the effect of group V and group VI transition metal carbide for the DRM reaction (Brungs et al., 1999). More specifically, they used molybdenum, tungsten, vanadium, niobium, and tantalum oxide that they converted to carbide prior to operating in dry reforming conditions. The different catalyst were tested at 950°C under a 8 bar pressure for period varying from 5 to 25 h and GHSV between 1 and 2 × 10^3^ h^−1^. Vanadium carbide showed conversion in the magnitude of 70% followed by a fast deactivation to 50% in 25 h. The H_2_/CO ratio was in the magnitude of 1 for the first hours of the reaction then slowly decreased to 0.7 after 25 h, which may be a sign that the catalyst is getting oxidized by carbon dioxide leading to the production of carbon monoxide. It could as well be a sign of carbon dioxide reduction to carbon monoxide using hydrogen thus lowering the ratio. The NbC catalyst started in the range of 60% CH_4_ conversion progressively lowering below 50% after 23 h. However, for this catalyst, the H_2_/CO ratio remained stable after 10 h at 0.8. TaC showed fast deactivation although the H_2_/CO ratio was close to one once the catalyst was operating only at 25–30% conversion of the methane feed. Mo_2_C however showed one of the best patterns for this work maintaining a methane conversion in the magnitude of 90% for 150 h. Brungs et al., also investigated the effect of temperature on their NbC catalyst. They tested at 1050 and 1100°C still under an 8 bar pressure with GHSV in the magnitude of 1.2 × 10^3^ h^−1^. At 1050°C, the conversion of methane was slightly higher than the conversion of CO_2_ around 90% whilst when temperature was increased to 1100°C, conversion was better for CO_2_ than for methane. This change led to a slow increase of the carbon monoxide produced in the process whilst the H_2_/CO ratio was slowly decreasing. Since the conversion of methane was as well increasing, it could be a sign of Equation (8) occurring thus lowering the H_2_/CO whilst increasing the CO_2_ produced.

Tsyganok et al., investigated on the effect of noble metals (ruthenium, platinum, and rhodium) supported on mixed magnesium and aluminum oxides (Tsyganok et al., 2004). The concentration of noble metals on the catalyst support was generally in the range of 2% wt. and operating temperature tested was of 850°C. In that work, the authors did not rely solely on dry reforming but used as well oxygen in their experiments to increase the oxidative potential of the medium. The feed ratio was therefore composed of CH_4_/O_2_/CO_2_/N_2_ = 35.2/16.2/3.18/45.8. Results, over a 5 h test, showed that both rhodium an ruthenium had good impact on the reaction allowing steady conversion of methane in the range of 95%. CH_4_ conversion on platinum decreased steadily down from 85 to 75% during the first 4 h of the tests after what it rose back to 80% during the fifth h. As for the H_2_/CO ratio, the rhodium catalyst was shown to be the most stable maintaining a ratio in the range of 1.85 for the whole duration of the experiment. The ruthenium catalyst stabilized after 3 h a little above 1.9 whilst the platinum catalyst dropped from a ratio of 2 to about 1.80. Although Tsyganok et al., work was not purely related to dry reforming, this approach has nevertheless a very interesting potential in terms of application. Since the dry reforming reaction is combined with partial oxidation of methane with oxygen, balancing the latter correctly with regards to the methane content may allow the reaction to be self-heated. This would avoid utilization of an outside source of energy to heat the system, which would be invariably the case for dry reforming with regards to its endothermic nature. Furthermore, the produce H_2_/CO ratio is closer to 2, which is of interest if the produced syngas is intended for catalytic conversion to fuel as an example. Bouarab et al., reported on a cobalt catalyst supported on silica that was modified by addition of magnesium oxide (Bouarab et al., 2004). Although the content of cobalt remained between 3 and 5% for all experiments, the amount of magnesium oxide added to the catalyst varied from 5 to 35% wt. The authors tested their catalytic system at 600°C and 1 atm using a 22 mL/min flow rate. At 600°C the equilibrium for the dry reforming reaction predicts that methane conversion should be of 42.7 % and results showed that adding magnesium oxide to the catalyst not only permitted to reach the equilibrium conversions but as well allowed maintaining the catalyst avoiding coke formation. When the authors did not use any MgO, the conversion of methane was originally of 41%, decreasing to 16.2% after 3 h. Coke formation for this catalyst was reported to be of 0.41% wt. after 3 h as well. Adding 5% of MgO to the mixture improved slightly the methane conversion to 42.3%, however, the deactivation was significantly higher after 3 h when the methane conversion reached 10%. Adding 10 of MgO had the same effect on methane conversion and deactivation. However, adding 35% of MgO to the catalyst (Co content was of 3.9% wt.) lead to a 42.1% conversion of methane that rose to 42.4% after 3 h, with no sign of deactivation. Furthermore, no carbon formation was reported for this specific catalyst. In all cases, conversion of CO_2_ was higher than anticipated by equilibrium ranging from 61 to 70% whilst equilibrium calculations at this temperature predicted 55.6% conversion. The best catalyst was then tested for a longer run reaching 30 h at steady state without any sign of deactivation. The H_2_/CO ratio for this part of the experiment was reported to be slightly above 1. Such an observation is rather intriguing since the conversion of carbon dioxide is reported to be higher than for methane. Choudhary et al., used the combined oxidative effect of carbon dioxide and water for their experiments where the catalyst was composed of neodymium and cobalt (Choudhary et al., 2005). Part of the article reported on tests that were performed at 850°C with a CO_2_ + H_2_O/CH_4_ ratio of 1.06. Using the same oxidizing/methane ratio, they varied the proportion of CO_2_/H_2_O and verified the impact on different aspect of the SDRM reaction. Lower concentration of CO_2_ led to higher H_2_/CO ratio, which is logical since steam was being the major oxidizing agent in the reaction. The same ratio deceased proportionally with the increasing amount of CO_2_ leaning toward a 1.5 H_2_/CO ratio. Methane conversion increased significantly until a CO_2_/H_2_O ratio of 1 was reached after what methane conversion still increased but more slowly. Choudhary et al., as well investigated their catalyst under PO-DRM conditions using O_2_/CH_4_ ratio of 0.45 and CO_2_/CH_4_ ratio of 0.14. Under these conditions, conversion of oxygen as well as methane remained steady in the range of 100 and 80% respectively. Conversion of CO_2_ started around 20% at 800°C and reached 70% at 900°C. Finally, increasing the gas hourly space velocity (GHSV) led to a reduction in conversion of both methane and carbon dioxide whilst oxygen remained close to 100%. However, the H_2_/CO ratio remained still around 1.6 for the different GHSV values. Wisniewski et al., reported on a iridium catalyst supported on a mixed metal oxide called CGO (Ce_0.9_Gd_0.1_O_2−x_) for the DRM in the 600–800°C temperature range (Wisniewski et al., 2005). The initial loading of Ir on the support was of 0.16% wt. Results shows that temperature, combined to the CH_4_/CO_2_ ratio had an impact on the production of water in the system where the maximum amount of water was obtained for a CH_4_/CO_2_ ratio of 0.67 at 750°C. Under classical dry reforming conditions (ratio of 1), the amount of water produced during the reaction increased slightly up to 2.5% from 600 to 700°C then decreased at 800°C. This phenomenon could be related to an hydrogen reduction of CO_2_ as depicted by Equation (8). The authors reported as well on the conversion of methane, which was the highest when a CH_4_/CO_2_ ratio of 1 was used. The optimal conversion of methane was in the range of 70% whilst conversion of CO_2_ under the same condition reached 80%. Wisniewski et al., also reported on the impact of modifying temperature and feed composition on the H_2_/CO ratio. Operation at a CH_4_/CO_2_ ratio of 1 at 800°C provided a H_2_/CO ratio of 0.9. Increasing the CH_4_/CO_2_ ratio up to 2 however provided a H_2_/CO ratio closer to unity. The fact that the ratio was below 1 is in agreement with the methane and carbon dioxide consummated during the reaction as well as the potential hydrogen-induced CO_2_ reduction. Increasing the amount of methane available would thus generate more hydrogen and therefore increase the ratio. Özkara-Aydınoğlu et al., reported on different mixtures of platinum (1% wt. maximum) and cerium (0–5% wt.) supported on zirconium oxides (Özkara-Aydınoğlu et al., 2009). The authors first reported on the impact of temperature on the DRM reaction ranging from 475 to 730°C. Amongst the 4 catalyst that were tested the 1% Pt combined with 1% Ce by coimpregnation was the catalyst that performed the closed to equilibrium calculations with an H_2_/CO close to 1 at maximum operating temperature. The maximum conversion of methane was observed at 730°C for most of the catalyst that were then tested for deactivation under this temperature for 5 h. Again, the 1% Ce and 1% Pt catalyst was shown to be the more constant only with a slight decrease in methane conversion over the 5 h duration of the test. The results also showed that there was a significant difference between the same catalysts depending on the type of impregnation. In that sense, the catalyst that was produced out of 1% Pt and 1% Ce by coimpregnation was more efficient than the same catalyst loading impregnated in two steps. Increasing the amount of Ce up to 5% did not provide better results as well.

Table 5 shows other metal, oxides or mixtures of element that can be beneficial for the DRM reaction. However, most of the reported work showed results inferior to the one involving nickel as main catalyst. The interaction between methane cracking and the reverse Boudouard reaction seems as well to influence the effect of many of the catalyst reported in Table 5. Metal carbides, as reported by Brungs et al. (2000), Brungs et al. (1999) were shown to be very efficient for the reaction, leading to the assumption that carbon occurrence on the catalyst may not be as detrimental as observed for other catalyst. However, higher temperatures used in those specific experiments may induce other reactions that allow a good balance between carbon formation and removal.

## Other conversion approaches

Heterogeneous catalyst is by far the most popular approach for the DRM reaction. However, a very important part of the reforming reaction is energy, which has to be sufficient enough to overcome the thermodynamic barriers leading to the oxidation of methane and the reduction of carbon dioxide. Energy can, on a chemical point of view, be seen as a mean to allow electron transfer or increase vibrations in molecules. Such mechanisms may be initiated via different other means than classical heat. This part of the review is thus dedicated to other approaches that were reported in literature as procedures promoting “unconventional” DRM reactions. Plasma technologies are amongst the most popular of the unconventional approached reported in literature. However, other technologies, often punctual or rarely investigated will as well be discussed below.

Table 6 below shows specific data that were obtained from open literature for the utilization of plasma for the DRM reaction. The configuration of the table has been changed in this specific case adding the power and flow values. Since DRM are often compared to SMR or ATR, the entering flow, conversions and power usage are very important aspects to quantify the potential of such techniques.

**Table 6:** **Dry reforming reactions reported in open litterature using different types of plasmas with regards to applied power (P)**.

**Plasma/Catalyst**	**Feed (mL/min)**	**P (W)**	**X_**CH4**_%**	**X_**CO2**_%**	**H_**2**_/CO**	**References**
Dielectric barrier discharge/Ni/γ-Al_2_O_3_	25	60	50	30	0.65	Tu and Whitehead, 2012
Dielectric barrier discharge/Ni/Al_2_O_3_	50	38.4	60	40	1	Wang et al., 2009a
Dielectric barrier discharge/Ni/Al_2_O_3_	250	50–80	28	26	n.d.	Wang et al., 2009b
DC thermal plasma/Ni/Al_2_O_3_	3.67 × 10^4^	9.6 × 10^3^	88.28	76.05	0.81^*^	Tao et al., 2008
Dielectric barrier discharge	500	500	35	20	0.5	Zhou et al., 1998
Gliding arc discharge	12,700	544	40	40	0.94	Bo et al., 2008
Kilohertz spark-discharge plasma	150	503	75	70	1.45^**^	Zhu et al., 2012
Glow discharge plasma	1000	69	95	80	1.2	Li et al., 2009
DC-Pulsed Plasma	90–180	135	55	45	1.1	Seyed-Matin et al., 2010
Arc-Jet Plasma	4000	1000	50	35	1.1	Hwang et al., 2010

*XCH_4_, Conversion of methane*.

*XCO_2_, Conversion of carbon dioxide*.

*H_2_/CO, Hydrogen to carbon monoxide ratio in the output gas*.

* *CH_4_/CO_2_, entering feed ratio was 0.67*.

** *CH_4_/CO_2_, entering feed ratio was 1.5*.

Tu and Whitehead reported on the effect of plasma using a (10% wt.) Ni/γ-Al_2_O_3_ calcined at different temperatures (300, 500, and 800°C) (Tu and Whitehead, 2012). Results shows that plasma has indeed a positive effect on the conversion of methane and even without any catalyst the experimental setup by itself allowed a maximum conversion of methane in the range of 50%. Better conditions were obtained at a 25 sccm entering flow and a 60 W discharge power. The authors as well investigated the effect of using the catalyst in the plasma reactor and found that the form of the catalyst had an impact on conversion. The reaction involving larger catalyst pellets led to a slight increase of methane conversion whilst CO_2_ remained at the same level. Tu and Whitehead also noticed that the effect of quartz wool in the reactor was as well beneficial for the reaction, which showed the best conversion both for methane and CO_2_. The calcination temperature of the catalyst had as well an impact on the conversion of methane since amongst the three catalysts that was investigated in that part of the manuscript, the one calcinated at 300°C showed the best activity increasing conversion of methane by 26%. However, effect on the conversion of methane decrease proportionally to the calcination temperature for the catalyst and the one that was treated at 800°C prior to the reaction did not show any gain in conversion as compared to the plasma without any catalyst. Wang et al., reported on the effect of low temperature plasma created by a dielectric barrier discharge (DBD) combined with a classical Ni/Al_2_O_3_ catalyst (Wang et al., 2009a). They investigated different types of configurations in their reactive system going from the catalyst being completely isolated from the DBD (mode A) to a system where the DBD and the catalyst were one next to the other (mode B) then a last one where they were assembled together (mode C). Results shows that mode A et mode B provided comparable results showing conversion of methane in the range of 40% for the highest operating temperatures (550°C). There were no sign of synergy between the catalyst and the plasma at higher temperature although a gap between the system with and without catalyst could be observed around 300°C. Mode C however, shows significant synergy between the DBD and the catalyst with a conversion gap for methane of 30% at the highest operating temperature. The higher conversions observed by Wang et al., were clearly a result of the synergic effect between the catalyst and the DBD using mode C configurations. However, one of the important aspects about this work is that it shows higher conversion of a higher feed at a lower energy input, which are good conditions in order to transfer such technologies at an industrial level. Wang et al., also reported on the impact of the catalyst bed on the DRM reaction using a system comparable to the one presented in their previous work (Wang et al., 2009b). In this work, not only they investigated the impact of fluidized bed but as well the impact of the total plasma power on the methane conversion. When working at room temperature, the authors observed that the impact of the fixed bed was more positive on the total conversion as compared to the fluidized bed. Conversions of 12% were achieved at a 80 W power for a fixed bed catalytic system whilst about 7.5% were achieved using a fluidized bed. Increasing the temperature at a fixed power for the plasma permitted to reach conversions close to 50% for the fixed bed system whilst close to 40% for the fluidized system. Increasing the operating temperature to 450°C whilst modifying the power of the plasma led to an increase of the conversion both for the packed bed and the fluidized bed catalyst although the latter remained significantly lower the first one. Furthermore, the conversion gap increased for the fluidized bed when operating at higher input power. As an example, the gap between conversions was of about 5% methane conversion at 35 W whilst it reached close to 10% at 50 W. Tao et al., investigated as well the combined effect of catalyst (a commercial nickel on alumina catalyst—Z107) and plasma on the DRM reaction (Tao et al., 2008). In their experimental system, the plasma was produced and fed directly at the top of a fixed bed where the catalyst was held. The authors tested various feed rates ranging from 1.5 to 2.5 m^3^/min with a constant input power of 9.6 kW using a CH_4_/CO_2_ ratio of 4/6. Results shows that the lowest feed flows (1.6 m^3^/h) produced conversion of methane in the range of 90% for experiments using plasma only and above 95% for experiments combining both plasma and catalyst. However, conversion decreased fast when increasing flow rate and dropped below 80% at 2.4 L/min for the experiments without catalyst and above 85% for experiments with the catalyst. As for CO_2_, its conversion was generally lower than that of methane although the catalyst was showed to have an impact as well on its conversion going from 80 to 85% at the lowest flow rates investigated in this work. High conversion for this work can be resulting both from the plasma as well as from the fixed bed reactor that was operated at 1000°C, which favors the thermodynamics of the dry reforming reaction. Therefore, it was rather predictable that conversion would be high, however, the combination of plasma and catalyst may have contributed to reduce the required energy per molecules of methane converted thus making this process more economical. Zhou et al., investigated the non-equilibrium plasma DRM reaction without the use of a complementary catalyst (Zhou et al., 1998). Many parameters were modified during this experiment including CH_4_/CO_2_ ratios, feed rates and power input. The authors showed that using 500 W DBD plasma, they were able to reach methane conversions in the magnitude of 30–35% and 20% for CO_2_. The authors reported as well that the wall temperature was of 80°C using a 1 atm pressure and the H_2_/CO ratio was around 0.5 when operating under these conditions.

Bo et al., studied the effect of gliding arc discharge (GAD) on the DRM reaction without any catalyst under a voltage ranging from 5.90 to 10.00 kV (Bo et al., 2008). The gasses, entering the reactor at a flow rate of 12.7 SL per minute were heated to 175°C prior to their contact with the GAD. Their system was tested under three different CH_4_/CO_2_ ratios of 0.5, 1, and 2. As far as CO_2_ is concerned, increasing the proportion of methane led to a decrease in conversion of CO_2_ going from close to 40% at 10 kV to nearly 25% at a two to one CH_4_/CO_2_ ratio. Conversion of methane was as well in the range of 40% both for ½ and 1/1 CH_4_/CO_2_ ratios although decreased to 30% when the CH_4_/CO_2_ ratio reached 2. Bo et al., also noticed ethylene and acetylene that formed as secondary during their process. Although both compound where found at low concentrations at a CH_4_/CO_2_ ratio of 0.5, they reached close to 10% under pure dry reforming conditions (ratio of 1) and increased further when methane was in excess. In some specific cases, the conversion of methane to acetylene reached close to 40% of the output gas (at a 5.90 kV voltage). Zhu et al., investigated the impact of kHz spark-discharge plasma with a rotary electrode on the DRM reaction with an emphasis toward the effect of pressure (Zhu et al., 2012). The authors expressed the energy provided to the system in terms of SEI (specific energy input) and quantified all their reacting conditions according to this specific energy. Using a fixed SEI value of 619 kJ/mol with an entering flow of 150 sccm and 60/40 methane on carbon dioxide ratio, they showed that conversion of methane increased proportionally to the pressure of the system. The conversion of methane was quantified at 70% at 1 bar, whilst increased at 72.5% at 1.5 bar, and 75% at 2 bar. Increasing pressure did as well increased conversion of CO_2_, which in all cases remained below the conversion of methane varying from 61 to 70% from 1 to 2 bar respectively. The H_2_/CO ratio started at 1.5 for the tests at 1 bar and decreased slightly to approximately 1.45 at 2 bar. These values were correlated to the production of acetylene, which occurred in this reactive system, which could explained the lower amounts of carbon monoxide as compared to hydrogen. As well, the authors used higher methane to carbon dioxide ratio, which can without any doubt explain why the H_2_/CO is significantly above unity.

Li et al., investigated on the impact of using glow discharge plasma at atmospheric pressure for the DRM reaction (Li et al., 2009). The system involved solely a plasma torch that was operated at about 69 W with a total entering flow of 1 L/min for both reactants. The authors investigated the effect of the CH_4_/CO_2_ feed ratio as well as the effect of the flow on conversion of both methane and carbon dioxide. Results for this experiment shows very efficient conversion of methane when operating at 1 L total entering flow per minute. Lowering the CH_4_ concentration with regards to CO_2_ leads to better conversion of methane but in most cases, even for pure dry reforming (equal amounts of CH_4_/CO_2_) the conversion were around if not above 90%. Hydrogen to carbon monoxide ratio was 1 for a 3:7 CH_4_/CO_2_ ratio whilst it climbed to 1.2 when using a 5:5 CH_4_/CO_2_ ratio. This may be a sign that the system induces carbon formation somewhere in the apparatus outside of the hot zone where the plasma was held. Increasing the amount of mL per unit of power per unit of time (referred to as ϕ by the authors and expressed in mL/min/W) showed that the high conversion that could be achieved at a higher unit of power per mL of CH_4_ were significantly declining when using larger volumes of methane. Therefore, going from 5 to 60 mL/W/min led to a decrease in methane conversion from 95 to 15%. Such observation could be resulting from the endothermicity of the dry reforming reaction. Furthermore, this increase also impacts on the H_2_/CO ratio, which reaches 2.6 when using a 60 mL/W/min feed-power ratio most probably a sign that methane cracking is not counterbalanced by any Boudouard reaction because of insufficient energy. Seyed-Matin et al., investigated on the impact of using DC-pulsed plasma for the DRM reaction without adding any catalyst (Seyed-Matin et al., 2010). They showed that, in accordance to the thermodynamics of the reaction, increasing the amount of energy invested per volume unit of reactant leads to higher conversions. Thus, conversion of methane reached 34% at 45 kJ/L whilst conversion of carbon dioxide showed lower conversion (about 20%). Increasing the amount of energy even higher (90 kJ/L) showed to have a positive impact on methane conversion that reached 55% whilst CO_2_ remained lower around 45%. At lower energy investment (between 6 and 12 kJ/L) the authors mentioned as well the production of acetylene in the mixture which was also observed by other researcher's work summarized in Table 6. Seyed-Matin et al., as well demonstrated the threshold in H_2_/CO ratio occurring at high flow when the energy invested to the mixture is between 5 and 10 kJ/L. Lowering the flow rate from 180 mL/min to 90 mL/min logically displaced this threshold which was not noticeable in the 50–10 kJ/L range. Modifying the CH_4_/CO_2_ ratio had positive effects on the conversion of both gasses when using a fixed pulsed of 600 Hz at a 6 kV voltage. Modifying the ratio also increased the production of acetylene whilst reducing the production of carbon monoxide whilst hydrogen production remained constant. This shows that under the actual conditions, the methane cracking reaction was not complete and that lesser volumes were actually oxidizing to carbon dioxide. Hwang et al., reported on the utilization of arc-jet plasma for the DRM reaction and studied different parameters including the electrical power invested in the reaction as well as the oxidizing ratio (Hwang et al., 2010). The authors noticed that for a fixed entering flow of 20 L/min (amongst which 2 L for CH_4_, 2 L for CO_2_, and 16 L of N_2_), increasing power from 500 to 1000 W led to an increased conversion of methane reaching 50% at 1000 W. Conversion of CO_2_ also increased with regards to time although it reached a slightly lower maximum (35%) as compared to CH_4_. The output gas was slightly in favor of hydrogen (H_2_:CO range of 1.10) and they as well noticed the occurrence of acetylene in the output gas which represented approximately 20% of the products. Modifying the CO_2_/CH_4_ ratio did not influence significantly the conversion for both gasses although the composition of the output gas was significantly influenced. Under strict dry reforming conditions (CH_4_/CO_2_ = 1), the H_2_/CO ratio was around 1 whilst increasing the ratio up to 2 produced a H_2_/CO below unity.

Table 6 shows the plasma technology could be used for the DRM reaction and amongst the different publications that were reviewed, combination of plasma and catalyst was shown to provide some of the best conversions reported. When using technology as plasma for the conversion of methane to syngas, one of the most important factors that have to be taken into consideration is the amount of energy invested per moles of methane. In order to make dry reforming competitive with actual reforming technologies, it has to represent an equivalent or lower power investment as compared to classical technologies. The rule of thumb used for the energy balance of ATR is usually to consider ¼ of the energetic value of methane to provide the heat necessary for the reaction. Since one mole of methane has a heating value of 802.27 kJ/mol, a dry reforming process should not invest more than 200 kJ/mol of methane converted. As an example, when using an feed of 25 mL/min combined with an energy input of 60 W, the energy provided to the system is approximately 8.5 times the energy value of methane entering the reactor keeping in mind the 50% conversion of methane as reported by Tu and Whitehead (2012). Increasing the flow rate to 50 mL/min and reducing power to 38.5 W keeping in mind the 60% conversion of methane provide a E_in_/E_CH4_ in the range of 2.25 calculated from the results published by Wang et al. (2009a). The second report by Wang et al., shows a reduction of this ratio to 1.26. The work reported by Tao et al. (2008) shows one of the best E_in_/E_CH_4__ ratio from Table 6 with 0.52, bested by Bo et al., that reached a ratio of 0.19 using their gliding arc technology (Bo et al., 2008) which is as well behind Li et al., and their glow-discharge plasma technology (Li et al., 2009) with a ratio of 0.12. Therefore, process reported by Bo et al. (2008) and Li et al. (2009) would have the good metrics in order to be scaled to larger processes according solely on the energy investments. However, plasma technologies by themselves are probably more expensive than classical fixed bed or ATR systems thus the initial investment may be larger. As well, it is not entirely sure at this point how does technology would behave at a larger scale.

In addition to plasma technologies, the microwaves-assisted processes are an example of unconventional processes investigated for the DRM reaction. Fidalgo et al., reported on the utilization of a microwave reactor combined with activated carbon that was used both as catalyst and as microwave receptor (Fidalgo et al., 2008). Amongst the minerals that they identified in the activated carbon a couple were as well used as heterogeneous catalyst in some of the work reported above in the first section of this review (TiO_2_, MgO, Na_2_O, CaO, Fe_2_O_3_, Al_2_O_3_, K_2_O). First test that the authors reported was in order to compare the impact of microwave on the reaction as compared to a classical electrical furnace. In both cases, operating temperature was of 800°C. Results show a faster deactivation for the system heated with the electrical furnace, which reached a starting conversion of methane of 75% whilst the microwave reactor allowed achieving a conversion of nearly 100%. Significant deactivation occurred after 90 min for the microwave reactor whilst it was observed only after 5 min in the other system. The highest conversion both for methane and carbon dioxide were obtained when operating at 700 and 800°C with 8 g of catalyst thus generating a GHSV (for methane) of 0.16 (L/g^*^h). In these conditions, carbon dioxide was slightly more concentrated in the feed (60%), which in both cases produced a H_2_/CO below unity (0.7). However, the system remained stable for both temperatures although at 700°C some minor deactivation was observed after 300 min. The same team reported on the utilization of slags from steel making combined to two different carbonaceous materials (activated carbon and metallurgical char) as catalyst for a microwave-assisted dry reforming reaction (Bermudez et al., 2012). The authors mentioned that the slags alone were not reactive toward microwave and could not be used without any support. Results from this work showed that activated carbon had (as reported in Fidalgo et al., 2008) a positive impact on the reaction when operating at 800°C. As well, metallurgical coke generated only low conversions and fast deactivation both for methane and CO_2_. Combining the sludges to the activated carbon however allowed a better longevity of the catalyst which remain stable after a slight deactivation. Furthermore, conversion tended to increase after 110 min of operation in the reactor. Combining the suldges with the metallurgical coke did as well improve performance although in that case the deactivation was more significant with regards to the slags combined to the activated carbon. Amongst the unconventional approaches reported for the DRM reaction, another was reported by Dahl et al., that used a solar-thermal aerosol flow reactor that uses the sun's energy to heat the reaction (Dahl et al., 2004). The authors used an High Flux Solar Furnace combined with a quartz tube that could reach operating temperatures above 1500°C. Results shows that operating at 1800°C, conversion of methane was in the range of 70% when using a 1 slpm feed of methane and carbon dioxide combined. Increasing the flow at the same operating temperature decrease conversion to about 60%. The authors reported as well that at the maximum operating temperatures, the power level of the furnace was approximately 9800 W.

Finally, recent work reported by Labrecque and Lavoie shows high conversions of methane using a fixed bed reactor induced with an electrical current (Labrecque and Lavoie, 2011). The authors showed that it was possible to convert most of the methane entering the reactor (1.70 × 10^−4^ mol/s) under steam/dry reforming conditions when using a steel wool catalyst and 400–440 W. The energy ratio (E_in_/E_CH4_) was in the range of 3.23, which is comparable to some of the less energy-efficient plasma technologies reported in Table 6. Although the energy balance of the system did not make it suitable for scale up at this specific point, it showed a new approach for the DRM reaction. Later, Banville et al. (2013) took the same reaction and investigated the effect of the catalyst. They showed that the steel wool was in balance with the iron oxide when the system was at steady state. Operating at temperature above 950°C with a CH_4_/CO_2_ ratio slighty superior to 1 allowed maintaining the catalyst whilst reducing this ratio below 1 led to a fast oxidation of the iron catalyst. As well the authors showed that even at a reducing ratio (above 1) the oxidation of the catalyst did occur when operating at temperatures below 850°C.

## Conclusion

Reforming technologies are gaining in importance around the world because of the availability and price of natural gas. The actual market price of 4.4 USD/GJ (before shipping) makes this carbon substrate one of the most affordable sources of energy actually available in the world. Methane can be used directly for combustion thus generating heat and/or power. However, it can be as well used for the production of longer chain alkanes through synthesis thus requiring a preliminary conversion to syngas. Syngas is a mixture of carbon monoxide and hydrogen produced from the reforming of natural gas. There is actually a wide variety of reforming technologies readily available on the market including autothermal reforming (ATR), steam reforming (SMR), dry reforming (DRM) or a combination of the latter. Whilst ATR uses oxygen (and/or water and or steam) to produce syngas, SMR used essentially steam whilst dry reforming uses carbon dioxide. Amongst all these reactions, dry reforming is the more thermodynamically unfavorable, however it has the advantage of using carbon dioxide as oxidizing agent. DRM technologies, although promising on an environmental point of view, are not operated at an industrial scale whilst the ATR and the SMR are. Other than the fact that the dry reforming reaction is highly endothermic as compared to the two others, the reaction often involves production of char that leads to deactivation of the catalyst. Dry reforming has the other downside of requiring large amounts of “pure” CO_2_, which cannot be industrially obtained easily except from a few specific technologies. As an example, isolating carbon dioxide from air would be very expensive (even if CO_2_ as a tipping fee) as well as isolating from thermal power plants. However, technologies as gasification or fermentation are technologies that produce significant volumes of high purity CO_2_ and could be good candidates for a first generation of industrial scale dry reforming processes.

Research and development in the field is very active and one of the most common catalysts used to this purpose is nickel supported on alumina. Since the latter is a common catalyst for classical reforming technologies it was extensively studied in dry reforming conditions hoping that it could be adapted to this process. The average operating time reported from literature was in the range of 20–40 h operation. However, most of the literature reviewed did not show any deactivation after this period. The nickel-based catalyst that showed the best potential was often catalyst that had small particles and crystallites. Such observation could be related to the potential formation of coke on the nickel particles that would be promoted when the nickel particle were close one to the other. The systems that involved the smallest and the most well distributed particles where the most efficient for reforming methane as well as the most resistant to deactivation. Mixtures of elements (still including nickel) were shown as well to have a positive impact both on methane conversion as well as deactivation. Alkali or alkaline-earth metals have shown to increase the lifespan of the nickel catalyst significantly, never however, a catalyst was reported for more than 350 h of operation. The most efficient and commonly investigated in this work were potassium and potassium oxides.

Other metals as well were reported to be efficient catalyst for the DRM reactions. Rare metals as Rh, Ru, and Pt were shown to generate high conversion rates for methane. However, the operating temperature where significantly higher than the work reported on nickel based catalyst. Equilibrium calculation have shown that increasing temperature above 1000°C should promote the DRM in any case, thus the efficiency of these catalyst can hardly be compared to the classical nickel catalyst since the operating temperatures were quite different in both cases. Other studies showed the positive impact of metal carbides (Mo_2_C, VC, NbC, and TaC) on the DRM reaction. These results may be a sign that the dry reforming reaction could possibility be separated into two distinctive reaction, one where the cracking of methane would occur and the other where the coke deposition would be oxidized by carbon dioxide. Equilibrium between both reactions would thus provide the same results as the pure dry reforming reaction. A hypothesis that could therefore be drawn on this reaction would be to select a metal that does induce a good cracking of methane without however being kinetically superior to the Boudouard reaction. The perfect balance between the two reactions would thus provide an effective system at equilibrium. This may be a reason explaining why in some situation the support on which the metal was fixed was so important and had a very distinctive impact on the reaction. Thus combining a well disperse element that promotes cracking of methane on a support that allows a better interaction with carbon dioxide may be a winning approach for the DRM reaction.

Utilization of plasma as well may be a good approach to induce high conversion of methane. Many plasma technologies have been discussed and many of them show a very positive energy balance. In some specific case, the reported energy consumption of the plasma represented about 15% of the calorific value of the methane thus making this process competitive to classical reforming technologies. However, plasma technologies are known to be expensive and scaling theses technologies may show to be tricky whilst ATR or SMR are well-know technologies that can be scaled accordingly. Furthermore, the utilization of electrical current to produce the plasma may be counterproductive on the carbon balance depending on how the electricity is produce in specific locations around the world. Using electricity made from fossil fuel (methane as an example) would contradict the environmental benefits that could be drawn from dry reforming. Finally, keeping in mind that the reaction may be divided into two aspects may lead to new technologies and some of these were shown to be very efficient using a very cheap catalyst (iron) and an electrical current.

With the actual tendency toward natural gas and shale gas exploitation around the world, reforming technologies will in the next year be investigated again and most probably new and optimized technologies will emerge. Amongst these technologies, dry reforming of methane has the advantage of using carbon dioxide as oxidizing agent, thus allowing its utilization as feedstock and especially when the syngas is intended for further conversion. Dry reforming not only requires a cheap source of methane, which can be available across the world but as well it requires a cheap and pure source of carbon dioxide, which may not be available in all locations. However, industries involved in the first and second-generation bio fuel production may be involved in such a process since in both case they produce large volume of pure carbon dioxide.

### Conflict of interest statement

The author declares that the research was conducted in the absence of any commercial or financial relationships that could be construed as a potential conflict of interest.

## Footnotes

^1^http://www.eia.gov/cfapps/ipdbproject/iedindex3.cfm?tid=3&pid=26&aid=2&cid=regions&syid=2011&eyid=2012&unit=BCF

## Abbreviations

ATR: Autothermal reforming system
DBD: Dielectric Barrier Discharge
DRM: Dry reforming of methane
GAD: Gliding arc discharge
GHSV: Gas hourly space velocity
HHV: Higher Heating Value
ICAD-HAT: Intercooled Aeroderivative Humidified Advanced Turbine
LHV: Lower Heating Value
PO-DRM: Partial oxidation and dry reforming of methane
SDRM: Steam dry reforming of methane
SMR: Steam Methane Reforming.
